# Jian-Wei Pan: building the quantum internet

**DOI:** 10.1093/nsr/nwy102

**Published:** 2018-09-21

**Authors:** Philip Ball

**Affiliations:** Writes for NSR from London

## Abstract

For many decades since its inception in the early twentieth century, quantum mechanics seemed to be an exotic and peculiarly non-intuitive kind of physics that applied to matter at the smallest scales: the laws that govern atoms, photons and subatomic particles. All our engineering, meanwhile, was dominated by the familiar rules of classical physics, in which objects have definite positions, trajectories and properties.

But, in the past several decades, scientists have started to harness quantum rules in practical technologies. In 1985, the physicist Richard Feynman suggested that computers governed by quantum rules might be capable of computations beyond the means of classical ones like those in use today. At much the same time, other researchers showed that information encoded in quantum states could be transmitted between a sender and receiver using a kind of encryption that could not be intercepted and read without that being detected. Quantum computers and quantum cryptography have now become central components of a real-world quantum-information technology that may soon find scientific, industrial and social uses.

These applications could be increasingly enabled by a global information network with quantum capability: a quantum internet. China is at the forefront of that enterprise, and one of the scientific leaders in this effort is Jian-Wei Pan of the University of Science and Technology of China in Hefei. Pan studied for his PhD with quantum-information pioneer Anton Zeilinger in Vienna before returning to China to implement these nascent technologies. In 2012, he won the International Quantum Communication Award and, in 2017, he was included in *Nature*’s annual list of the ‘ten people who mattered in science’ over the past year. That July, he and his colleagues reported ‘quantum teleportation’ of photons from a ground-based station to a satellite 1400 km away.

*NSR* recently interviewed Professor Pan about the current achievements and future prospects for quantum-information technologies.

**NSR:** The quantum internet is becoming a popular buzzword, but what will it mean?

**Pan:** As we know, the internet is a global system to transfer, process and store classical information. The quantum internet is the equivalent for quantum information. The first practical task for a quantum internet may be sharing secret keys globally with unconditional security [that is, they are absolutely tamper-proof]. Quantum bits (qubits) and quantum entanglement [the interdependence of states of the qubits] will be the basic resources of the quantum internet. Various quantum-information tasks can be realized in this system, such as quantum teleportation of information between any nodes, distributed quantum computation and high-precision quantum metrology.

**NSR:** What are the underlying principles of quantum physics that a quantum internet would use?

**Pan:** The fundamental principle that quantum-information theory is built on is *quantum superposition*. Superposition allows a qubit to represent not just the 0 and 1 of classical bits but also any intermediate state. Classically, a bit is either in state 0 or 1. But in the quantum world, a qubit can be in a superposition of 0+1, or another different combination of 0 and 1, simultaneously. A basic principle is that no single measurement is sufficient to reveal all the information. This leads to the quantum non-cloning theorem, which dictates that an unknown quantum state cannot be copied precisely. When a quantum system consists of two or more qubits, superposition becomes entanglement and makes the state space grows exponentially large. The uncertainty principle and the non-cloning principle are the cornerstones of quantum communication and quantum computation, as well as of the quantum internet.

**Figure 1:**
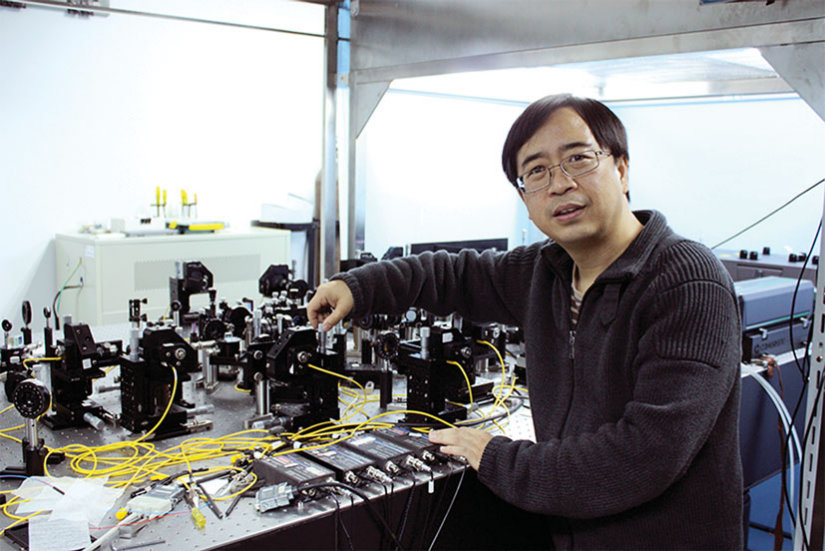
Jian-Wei Pan, professor of the University of Science and Technology of China (*Courtesy of Professor Pan*).

Quantum teleportation is a way to transfer an unknown quantum state from one particle to another at a distant location, without sending the original particle itself. Assume that a pair of entangled particles, denoted a and b, are exchanged between locations A and B. Then let's say an unknown qubit c needs to be transferred from A to B. To effect teleportation, at A we perform a collective measurement on a and c, followed by a certain operation (depending on the result of that collective measurement) on b. Then the (unknown) information will be ‘destroyed’ in c and ‘teleported’ to b. But B needs the information gained that A gained in the a+c measurement in order to make any sense of the information teleported to b. And that can only be sent classically, no faster than the speed of light. In other words, although the collapse of entanglement that sends the state of c onto b happens instantaneously, quantum teleportation can’t be used to transmit information faster than light.

**NSR:** What are the major technologies that are needed for a quantum internet? Could the existing fibre-optic and satellite infrastructure support it, or is new infrastructure needed, for example?

**Pan:** Generally, the quantum internet will consist of nodes that process and store quantum information, and channels that transfer quantum information. The nodes are complicated and highly dependent on the specific task at hand. For example, quantum key distribution (QKD) is the first practical application of quantum information. In China, the Beijing-Shanghai Backbone Network is already built and put into use. A QKD terminal must be capable of producing, manipulating and measuring single photons.

The best particle for transferring qubits (making ‘flying qubits’) is the photon. It has the big advantage that laser communication is now quite mature and there is already a mass of optical infrastructure can be used—so yes, existing fibre-optic channels can indeed be used. However, big modifications are still needed for the quantum internet, especially at the nodes. As a satellite is extremely hard to modify, we believe that new satellites are necessary [tailored to quantum communication and processing]. There are some cases where quantum payloads could be added to existing scheduled satellites, for example with GPS satellites or the European Galileo satellite.

**NSR:** Is this mainly about making internet telecommunications more secure, or are there other benefits that could come from encoding information using quantum rules?

**Pan:** The near-term target is indeed to make telecommunication more secure. But quantum information can do more than this. We believe that a practical quantum computer can be built within a few decades. Through the quantum internet, cloud quantum computation will be a basic resource in the future.

**NSR:** What has been achieved so far, and what would be the future milestones in creating a system like this?

**Pan:** We have built the Quantum Secure Communication Beijing-Shanghai Backbone Network. As the world's first long-distance quantum-secured communication route, it was put into service on 29 September 2017. The inter-city quantum communication line, which measures over 2000 km and comprises 32 relay stations, connects the cities of Beijing, Jinan, Hefei and Shanghai. Data can be transferred through the network with information-theoretical security. More backbone and inner-city networks are being planned and will be built. We have launched the first quantum science satellite, Micius, on 16 August 2016.

The near-term target is to make telecommunication more secure.

—Jian-Wei Pan

The quantum internet of the future might be completely different from what we imagine now.

—Jian-Wei Pan

QKD has been performed between Micius and several ground stations in China, including Beijing, Delingha, Nanshan, Graz in Austria and Tenerife in Spain. With the help of the Beijing-Shanghai Backbone, intercontinental QKD has thus been realized via Micius. More satellites will be launched to constitute a global network in the future.

**NSR:** Do we yet have a full understanding of the principles behind a quantum internet, making it ‘just’ a matter of engineering? Or do you think we might yet discover new things that quantum physics makes possible for information technology, in the way that we have already for cryptography and computing?

**Pan:** We don’t have a full understanding even of the principle behind the very basic phenomenon of quantum superposition. However, that doesn’t hamper the applications of quantum mechanics with the knowledge we already have. This knowledge makes building a QKD network seems like an engineering project. However, even in this relatively mature domain, new concepts continue to emerge. For future techniques such as quantum computation, we still know too little. The quantum internet of the future might be completely different from what we imagine now. I think this is the magic of science.

**NSR:** Does a quantum internet necessarily have any connection to the development of quantum computers, or is it wholly separate? Might a quantum-based telecommunication system enable distributed quantum computing, for example?

**Pan:** Yes, it will. I believe that quantum computers will become a crucial part of the quantum internet. Due to their high construction cost, quantum computers will be expensive resources, and so will offer a public service only via the quantum internet—at least in the early stages. In this scenario, users will access quantum computers through the quantum internet, uploading tasks and downloading results by transferring qubits. This is the concept of cloud quantum computation.

**NSR:** What kind of investment is being made internationally in developing a quantum internet? Will it require trans-national cooperation? Where are the major centres of research internationally?

**Pan:** The quantum internet involves lots of theory and technology. For the short-term target of building a quantum internet for global secure communication, we will have to consider the imperfections of realistic devices. We need better protocols to minimize the influence of engineering imperfections. We need cheaper and better devices that offer improved performance and can work in extreme environments. We need quantum channels that are widely distributed and easy to use, including both fibre and satellite links. I can’t imagine that any country can meet these challenges alone, without international cooperation. We have a great team here in China, and there are also very good teams all over the world. Already there is very good international cooperation on these problems.

**NSR:** China has been a leader in this area. Does the Chinese government see this technology as a particularly important one for future growth of the economy, finance, business and so forth?

**Pan:** Yes, we have received strong support from the Chinese government. As I mentioned above, we have built and put into service the Beijing-Shanghai Backbone Network, and currently

real-world applications by banks, securities and insurance are being trialled. More than 150 users are now using our Backbone for secure information transfer. As I also mentioned, we have launched the first quantum science satellite, Micius.

**NSR:** Where do you see this technology being in, say, 20 years time?

**Pan:** The cost of QKD will be dramatically reduced. Devices for QKD will be miniaturized and become suitable for personal use. QKD will be a common technique for encryption and be widely used in daily life. Quantum computers or quantum simulators will be built and run as public services for certain tasks, just as supercomputers are today. I don’t think that a universal [general-purpose] quantum computer that can factorize a large number, of say 2048 bits, will be built by that stage, we will already know what such a universal quantum computer should look like and what we still need to do in order to build one.

**NSR:** Who or what has been your source of inspiration in this field? Would you recommend it as an area for young researchers to enter?

**Pan:** I believe both foundational aspects of quantum mechanics and possible practical applications are very important issues. The original motivation for me to be an experimentalist was to perform fundamental tests of the laws of quantum mechanics, to understand how and why it differs from classical physics. However, the possible practical applications of quantum mechanics also attract me deeply, since they can make a real difference to our lives.

With the invention of the internet, we have entered the information age. The quantum internet provides another rare opportunity to truly change the world. It would enable a scientific revolution. I will spare no effort to recommend that smart young researchers engage with this area.

